# Legal analysis of South Korean cosmetic filler litigations for safer medical practices

**DOI:** 10.1038/s41598-024-63845-8

**Published:** 2024-06-10

**Authors:** Daihun Kang, Seung Eun Hong

**Affiliations:** Department of Plastic and Reconstructive Surgery, Ewha University Seoul Hospital, Ewha Womans University School of Medicine, 260, Gonghang-Daero, Gangseo-Gu, Seoul, 07804 Republic of Korea

**Keywords:** Cosmetic filler injections, HA filler, Medical malpractice, Complications, Violation of informed consent, Medical negligence, Biotechnology, Anatomy, Health care, Medical research, Risk factors

## Abstract

Cosmetic filler injections have gained popularity in recent years, but the rise in complications has led to an increase in legal disputes. This study analyzes civil court rulings related to cosmetic filler injection lawsuits in South Korea from 2007 to 2023. A retrospective case analysis was performed using a systematic database search, and a mixed-methods approach was employed for data analysis. The study examined 27 cases, revealing a high rate of liability findings against medical practitioners. Skin necrosis and blindness were the most common complications, and intravascular filler injection was recognized as negligence. Violation of informed consent was found in most cases, with mean compensation awards of ￦193,019,107 KRW ($142,831 USD) for first instance cases and ￦81,845,052 KRW ($60,564 USD) for second instance cases. The findings emphasize the importance of practitioner awareness, adherence to precautionary measures, and proactive prevention and management of complications. Collaboration among stakeholders is crucial for developing strategies that prioritize patient safety and minimize legal disputes in the aesthetic medicine industry. This study provides valuable insights for enhancing medical practices and safeguarding patient well-being in the field of cosmetic filler injections.

## Introduction

The rapid ascent of cosmetic filler injections as a popular aesthetic treatment has been a defining feature of the past decade^1,2^. With the promise of enhancing appearance and reversing the signs of aging, these minimally invasive procedures have attracted a growing number of individuals seeking cosmetic improvements^3,4^. However, the rise in popularity has been paralleled by an notable increase in complications and legal disputes associated with filler treatments^5^. This trend is particularly evident in South Korea, a country renowned for its advanced aesthetic medicine industry, where cases of medical malpractice and patient dissatisfaction related to filler injections have led to a surge in civil lawsuits^6^.

While generally considered safe, filler injections are not without inherent risks. Serious complications, such as skin necrosis, blindness, and even stroke, can occur when the filler substance is inadvertently injected into blood vessels^7,8^. These adverse events not only cause significant physical and psychological harm to patients but also result in significant legal and financial consequences for medical practitioners^9^. Moreover, the increasing incidence of filler-related complications has broader implications for public health, including rising healthcare costs, reduced quality of life for affected individuals, and erosion of trust in the medical profession^10^.

Despite the growing concerns surrounding filler-related complications and legal disputes, there remains a paucity of research examining the legal aspects of these cases in South Korea. A thorough analysis of court judgments pertaining to filler injection lawsuits can provide invaluable insights into the types of medical accidents, the criteria used by courts to determine negligence, and the standards employed in awarding damages. Furthermore, such an analysis can illuminate the complex legal issues surrounding filler procedures and contribute to the development of strategies and policies aimed at enhancing patient safety and preventing medical malpractice.

To address this knowledge gap, the present study seeks to conduct a comprehensive analysis of civil court rulings related to filler injection lawsuits in South Korea from 2007 to 2023. By thoroughly examining the characteristics of these legal cases, including the nature of complications, the reasoning employed by courts in determining negligence and liability, and the criteria used in awarding damages, we seek to provide actionable insights that can guide medical professionals, legal experts, and policymakers in their efforts to improve the safety and quality of care in aesthetic medicine.

The ultimate goal of this study is to contribute to the protection of patient rights and the prevention of medical accidents in the field of cosmetic filler injections. We anticipate that the findings of this research will serve as a foundation for developing evidence-based guidelines, informing medical education and training programs, and guiding policy decisions to foster a safer and more trustworthy environment for patients undergoing these procedures. By shedding light on the legal landscape of filler complications in South Korea and their broader societal impact, we hope to stimulate further research and collaboration among medical, legal, and regulatory stakeholders to address the challenges posed by this growing public health concern.

### Key legal terms

To ensure clarity for readers, we provide definitions for key terms used throughout this manuscript:

- Negligence: Failure to take proper care in doing something, resulting in damage or injury to another.
- Informed Consent: A process by which a patient is informed about the potential risks, benefits, and alternatives of a procedure and consents to it.
- Liability: Legal responsibility for one's actions or omissions.
- Unlawful: Actions that are contrary to legal standards and regulations governing medical practices.
- Civil Litigation: A legal process in which a civil lawsuit is resolved in a court of law.
- Compensation: Monetary award given to a plaintiff to compensate for loss or injury.
- Duty of Care: A legal obligation of healthcare professionals to adhere to a standard of reasonable care while performing any acts that could foreseeably harm patients.

## Methods

### Study design and data collection

This retrospective case analysis examined civil lawsuits related to cosmetic filler injections adjudicated in South Korean courts from 2007 to 2023. A comprehensive database search was conducted using the National Law Information Center (law.go.kr)^11^, a centralized legal information database managed by the South Korean Ministry of Government Legislation, and the case law search websites “Big Case^12^” and “Case Note^13^”.

The search terms included "filler", "cosmetic filler", "filler injection", and the names of various commercially available fillers in South Korea, such as “Restylane”, “Juvederm”, “Yvoire”, “Perfectha”, “The Chaeum”, “Radiesse”, “Cleviel”, and “Sculptra”. Terms related to medical professionals, such as "plastic surgeon", "dermatologist", and "physician", were also used.

Stringent selection criteria were applied to include only civil rulings pertaining to complications arising from cosmetic filler injections performed by licensed medical professionals. Cases involving filler injections, either performed illegally by unlicensed practitioners or accompanied by other surgeries or treatments on the same area, or primarily focused on conflicts between insurance companies and defendant medical professionals were excluded. Cases dealing with procedural matters, such as requests for jurisdictional transfer, were also omitted.

The research team, composed of legal experts and medical professionals, employed a systematic approach to document retrieval and assessment. The combination of official government databases and legal search engines ensured a thorough and comprehensive review of relevant cases.

### Data analysis

The analysis framework was predicated on a dual approach: qualitative analysis to unravel the thematic patterns and legal reasoning in court rulings, and quantitative analysis to assess compensation trends and the frequency of specific complications cited. The analysis of compensation trends is crucial as it highlights the financial implications of malpractice and the importance of adhering to medical standards to avoid litigation. Data points such as filler type, injection site, nature of complications, legal grounds for disputes, compensation outcomes, the gender of the plaintiff, and the location of the lawsuit were meticulously cataloged and analyzed. This methodology facilitated a granular understanding of the correlation between procedural specifics and legal outcomes.

### Ethical considerations

Given that this study is based on a sampling of legal documents available in the public domain and the absence of direct patient involvement, formal ethical clearance was not required. Nonetheless, the research protocol was meticulously designed to adhere to the highest standards of ethical integrity, including the principles outlined in the Declaration of Helsinki. Measures were taken to ensure anonymity and confidentiality, with all personal identifiers removed and data aggregated to protect the identities of individuals referenced indirectly through court documents. All analytical processes were conducted under the aegis of these ethical research principles, safeguarding the dignity and rights of the individuals involved.

## Results

### Litigation overview

- We analyzed a total of 27 cases, including 22 first instance cases and 5 s instance cases. First instance cases refer to initial trial court decisions, while second instance cases pertain to appellate court rulings.
- In the first instance, patients won 18 cases and doctors won 4 cases.
- In the second instance, patients won 5 cases and doctors won none.
- The regional distribution of lawsuits was as follows (Table 1)
- In the first instance, there were 16 cases in Seoul, 3 in Busan, 1 in Daejeon, 1 in Ulsan, and 1 in Changwon, totaling 22 cases.
- In the second instance, there were 4 cases in Seoul and 1 in Ulsan, totaling 5 cases.
- The gender ratio of plaintiffs (Table 1)
  - In the first instance was 2 males, 5 females, and 15 unknowns.
  - In the second instance, it was 1 male, 1 female, and 3 unknowns.
- The types of fillers used were as follows (Table 2)
  - In the first instance cases, out of a total of 22 cases, hyaluronic acid (HA) was used in 14 cases, calcium hydroxyapatite in 1 case, and a combination of HA and dextranomer in 1 case. The filler type was unknown in 6 cases.
  - In the second instance cases, out of a total of 5 cases, HA was used in 3 cases, and the filler type was unknown in 2 cases.
  - There were no cases involving calcium hydroxyapatite or HA/dextranomer combinations in the second instance.

**Table 1:** Gender distribution of plaintiffs in different regions with respect to legal instances.

Region and gender of plaintiffs	Capital	Non-capital	Male	Female	Unknown
1st instance	16	6	2	5	15
2nd instance	4	1	1	1	3

**Table 2:** Filler composition across court instances.

Filler component	First instance cases	Second instance cases
Hyaluronic acid (HA)	14	3
Calcium hydroxyapatite	1	0
HA & dextranomer	1	0
Unknown	6	2

Compensation and Consolation Money Analysis (Table 3).

- The average total compensation was ￦193,019,107 KRW ($142,831 USD) for 18 first instance cases and ￦81,845,052 KRW ($60,564) for 5 s instance.
- The average compensatory damages were ￦208,813,674 KRW ($154,519 USD) for 12 out of 18 first instance cases and ￦76,728,250 KRW ($56,778) for 3 out of 5 s instance cases.
- In all cases where compensatory damages were awarded, a violation of the duty to explain was recognized, and consolation money was additionally calculated.
- Consolation money was awarded in 18 out of 22 first instance cases and in all 5 s instance cases.
- The average consolation money was ￦26,470,588 KRW ($19,588) in the first instance and ￦28,700,000 KRW ($21,238) in the second instance.
- The exchange rate used for USD conversions is based on the rate as of April 10, 2024 (1 USD = 1,351.38 KRW).

**Table 3:** Compensation and consolation money analysis.

Instance	Total cases	Winner	No	Average total compensation	Number of damage compensation	Average damage compensation	Number of emotional distress compensation	Average emotional distress compensation
First	22	Patient	18	￦193,019,107 ($142,831)*	12	￦208,813,674 ($154,519)*	18	￦26,470,588 ($19,588)*
		Doctor	4					
Second	5	Patient	5	￦81,845,052 ($60,564)*	3	￦76,728,250 ($56,778)*	5	￦28,700,000 ($21,238)*
		Doctor	0					

*All USD amounts calculated using the exchange rate on April 10, 2024 (KRW, US dollars).

## Complication analysis

### Filler injection sites (Table 4)

**Table 4:** Injection sites and associated litigation.

Filler injection site	First instance cases	Second instance cases
Nose	15	4
Nasolabial fold	6	1
Forehead	1	0
Lower eyelid	3	1
Penis	1	0

Overlaps in filler injection sites were included in the count.

The most common site for filler injections leading to litigation was the nose, with 15 cases in the first instance and 4 cases in the second instance. The nasolabial fold was the second most common site, with 6 cases in the first instance and 1 case in the second instance. Other injection sites included the forehead (1 case in the first instance), lower eyelid (3 cases in the first instance and 1 case in the second instance), and penis (1 case in the first instance).

### Type of complications (Table 5)

**Table 5:** Complications leading to litigation.

Complications	First instance cases	Second instance cases
Blindness (right)	3	1
Blindness (left)	5	0
Skin necrosis	11	3
Stroke	3	0
Inflammation	2	1
Nodule	3	1

*Each complication occurrence was counted separately, including cases with multiple complications.

Among the various complications that led to litigation, skin necrosis was the most prevalent, occurring in 11 cases in the first instance and 3 cases in the second instance. Blindness was the second most common complication, with a total of 8 cases (3 in the right eye and 5 in the left eye) in the first instance and 1 case (right eye) in the second instance. Other complications included cerebral infarction (3 cases in the first instance), inflammation (2 cases in the first instance and 1 case in the second instance), and nodule formation (3 cases in the first instance and 1 case in the second instance). It is important to note that some cases involved multiple complications, and each complication was counted separately.

## Analysis of medical negligence recognition

### Cases of recognized liability

The doctor's liability for damages was recognized in 15 out of 22 first instance cases (68.2% of total first instances) and 3 out of 5 s instance cases (60.0% of total second instances) when the filler was injected into blood vessels, causing skin necrosis, blindness, or cerebral infarction (Table 6).

**Table 6:** Recognition of liability for damages in first and second instance cases.

Liability for damages	First instance	Second instance
Recognized	15 (68.2%)	3 (60%)
Not recognized	7 (31.8%)	2 (40%)
Total cases	22 (100%)	5 (100%)

### Key legal foundations in recognized liability cases

#### Mitigating the burden of proof

In 8 first instance cases (36.4% of total 1st instances, 53.3% of recognized liability in 1st instances) and 2 s instance cases (40% of total 2nd instances, 66.7% of recognized liability in 2nd instances), the Courts applied the principle of mitigating the burden of proof in medical malpractice lawsuits, considering the difficulty for victims to prove causation. If the victim proves indirect facts that can be viewed as a result of medical negligence, negligence is presumed unless the medical professional proves otherwise.

#### Intravascular filler injection as negligence

In 6 first instance cases (27.3% of total, 40.0% of recognized liability in 1st instances), the mere entry of filler into blood vessels was considered negligence. Courts have stipulated that doctors should pretreat with vasoconstrictors, perform regurgitation, use blunt-tipped cannulas, and avoid high-pressure single-shot injections. Failure to follow these precautionary measures and the occurrence of intravascular filler injection constitutes a breach of the standard of care and medical malpractice.

#### Illegal filler injection to minors

In 2 cases (1 first instance case, 1 s instance case), the doctor's liability was recognized as 90% when a filler was illegally injected into a minor.

### Cases of unrecognized liability

#### Subjective dissatisfaction with cosmetic results

In 1 first instance case (4.5% of total, 14.3% of exempted in 1st instances), subjective dissatisfaction with shape or form was not considered a basis for alleging negligence.

#### Complications within acceptable range

In 1 first instance case (4.5% of total, 14.3% of exempted in 1^st^ instances), pain and scarring in the nose within the range of general complications did not presume negligence based solely on the occurrence of sequelae.

#### Unlawfulness of imposing the burden of proving no negligence

In terms of Korean law, 'unlawful' denotes actions that are contrary to the legal standards and regulations governing medical practices. In 3 first instance cases (13.6% of total, 42.9% of exempted in 1st instances), it was ruled unlawful to impose the burden of proving no negligence on the doctor when the probability of presuming negligence was insufficient, such as infection after filler injection into the glans penis, scarring and deformation after nasal filler injection, and tissue necrosis and blindness after forehead and nasolabial fold filler injection.

Similarly, in 1 s instance case (20.0% of total, 50% of exempted), the court found it unlawful to place the burden of proving no negligence on the physician when the likelihood of presuming negligence was inadequate.

#### Lack of proof of violation of duty of care

In 1 s instance case (20% of total second instances, 50% of exempted cases in the second instances), the claim was dismissed due to a complete lack of proof of violation of the duty of care when granulomas occurred after periorbital and nasolabial fold filler injection.

#### Omission of judicial reasoning in small claims cases

In 2 first instance cases (28.6% of exempted in 1st instances), the reasons were not documented due to Article 11–2, Paragraph 3 of the Small Claims Act, which allows the judge to omit such details in minor civil cases. This provision led to the absence of explicitly stated reasons in the judgments.

## Doctor's liability ratio analysis

In the first and second instance cases where the doctor's liability for damages was recognized, the mean percentage of liability was 55.6% with a standard deviation of 41.3% (Table 7). In the first instance cases, the mean percentage of liability was 56.4% (SD 41.3%), while in the second instance cases, the mean percentage of liability was 48.0% (SD 43.8%). These findings suggest that when a doctor's negligence is acknowledged, a substantial level of liability for damages is imposed. However, there were significant variations in the liability ratio among cases, with some cases recognizing 100% of the doctor's responsibility, while others assigned 90%, 80%, 70%, 50%, 0%, or an unknown percentage.

**Table 7:** Doctor's liability ratio in first and second instance cases.

Liability ratio (%)	First instance cases	Second instance cases	Total (%)
100	1	0	1
90	4	1	5
80	5	1	6
70	2	1	3
50	1	0	1
0	8	2	10
Unknown	1	0	1
Mean ± SD	56.4 ± 41.3	48.0 ± 43.8	55.6 ± 41.3

Cases with unknown liability ratios were excluded from the mean and standard deviation calculations.

When the doctor's liability for damages was recognized in the first instance, the mean liability ratio was 81%. Due to the fundamental civil law principle of equal apportionment of damages, there was only one case in the entire dataset where 100% liability was assigned to the physician. Factors such as administration of enzymes, antihistamines, steroids, antibiotics, and NSAIDs after filler procedures were all considered as mitigating factors in limiting liability, regardless of their medical effectiveness or indications. Transfer to higher-level medical institutions and frequent patient visits by the treating physician while admitted at other facilities were also recognized as liability-limiting factors. Pre-existing patient factors, such as prior rhinoplasty surgery resulting in scarring, which increased the likelihood of intravascular filler injection, were all considered as mitigating circumstances.

Moreover, cases where the patient consumed alcohol and smoked despite the doctor's precautionary advice or instances where the patient took a shower on the second day after the procedure, leading to infection, were also regarded as factors that reduced the defendant's liability. However, with the exception of one case with 50% liability and one case with 100% liability, all other cases fell within the 70–90% range.

## Violation of the duty to explain and compensation for damages

In the first instance, a violation of the duty to explain was recognized in 18 out of 22 cases (81.8%). In the second instance, all 5 cases (100%) acknowledged the breach, and compensation for damages was ordered (Table 8). When the duty to explain is violated, the patient's right to self-determination is infringed upon, resulting in an order to compensate for the resulting mental distress. The scope of explanation was found to be extremely broad, requiring not only disclosure of simple nodule formation, foreign body sensation, infection, and pain but also all possible complications that may arise from intravascular filler injection, such as skin necrosis, blindness, and stroke. There were many cases where compensation for damages was ordered due to the lack of explanation about blindness or, in particular, stroke, even though skin necrosis was explained. The prevailing judgment of the South Korean courts was that regardless of how low the probability of occurrence may be, if the complication is severe, it must be explained in advance to avoid infringing upon the patient's right to self-determination. In the 18 first instance cases, the average amount of compensation for damages was ￦25,277,778 won (approximately $18,705 USD). In the second instance cases, the average compensation was ￦28,700,000 won (approximately $21,238 USD).

**Table 8:** Consolation money (solatium) in first and second instance cases.

Consolation money (solatium)	First instance	Second instance
Number of cases awarded	19 (86.4%)	5 (100%)
Average amount	￦25,277,778 KRW	￦28,700,000 KRW
	($18,705 USD)	($21,238 USD)
Standard deviation	￦29,287,474 KRW	￦45,983,149 KRW
	($21,672 USD)	($34,027 USD)

The USD amounts are based on the exchange rate of 1,351.38 KRW to 1 USD, which was the approximate rate on April 10, 2024.

However, there were cases where the physician's breach of duty of care was not recognized, and compensation for damages was not ordered, but consolation money (solatium) was still awarded. In the first instance, there were 3 such cases, and in the second instance, there were 2 cases. In all these cases, only a violation of the duty to explain was acknowledged without any negligence, and the severity of the breach was deemed to be not significant, resulting in relatively lower amounts of consolation money. In the first instance, there was one case of 10 million won ($7,399 USD), and two cases of 5 million won ($3,699 USD). In the second instance, there was one case of 1.5 million won ($1,109 USD) and one case of 5 million won ($3,699 USD based on the exchange rate as of April 10, 2024).

## Discussion

The present study offers a comprehensive analysis of civil litigation related to cosmetic filler injections in South Korea from 2007 to 2023. The findings provide valuable insights into the nature of these legal disputes, the factors influencing judicial decisions, and the broader implications for medical practice, legal frameworks, and public health policies.

The geographical analysis of cosmetic filler-related lawsuits indicates a pronounced clustering in major urban centers, with Seoul registering 16 cases at first instance and 4 at second instance. This concentration may be reflecting the intense competition among practitioners in the above region^14^. In the drive to attract patients, the emphasis on quantity over quality can inadvertently lead to less cautious application of procedures. Such an environment may inadvertently increase the incidence of complications and, consequently, litigation^1^. It's crucial for the medical community to acknowledge that the pressure to keep pace with competitors should not compromise the meticulous preparation and execution of filler injections^15^. Ensuring that patients are well-informed and that procedures are carried out with due diligence is paramount to prevent adverse outcomes, and it is the responsibility of each practitioner to uphold these standards of care to maintain the integrity of the aesthetic medicine field.

A significant aspect of our findings is the predominance of complications related to Hyaluronic Acid (HA) fillers over other types. HA fillers have gained popularity in South Korea due to their perceived safety, versatility, and reversibility^16^. The ability to dissolve HA fillers using hyaluronidase in case of adverse events has contributed to their widespread use^17^. However, the high prevalence of HA fillers in the market has also led to a greater number of reported complications associated with these products^8^.

The preference for HA fillers among both practitioners and patients can be attributed to several factors. First, HA fillers have a unique capacity to attract and retain water molecules, which contributes to their volumizing effect and natural-looking results^18^. The hydrophilic nature of HA allows the filler to integrate seamlessly with the surrounding tissues, creating a smooth and cohesive appearance^4^. This property also helps to maintain skin hydration and promote a more youthful and supple look^16^.

Moreover, the cross-linking technology used in modern HA fillers has greatly improved their longevity and stability compared to earlier generations of fillers^18^. The cross-linking process creates a more robust and viscous gel that can resist degradation and maintain its shape for an extended period^19^. This advancement has made HA fillers more predictable and cost-effective for patients seeking long-lasting results without the need for frequent touch-ups^18^.

Another advantage of HA fillers is their versatility in treating a wide range of facial concerns^16^. From fine lines and wrinkles to deep folds and volume loss, HA fillers can be used to address multiple signs of aging in a single session^20^. The variety of HA filler formulations with different particle sizes, cross-linking densities, and rheological properties allows practitioners to tailor treatments to the specific needs of each patient and to target different areas of the face with precision^18^.

Furthermore, the reversibility of HA fillers provides a safety net for both patients and practitioners^21^. In the event of an unsatisfactory outcome or a rare complication, the effects of HA fillers can be quickly and effectively reversed using hyaluronidase injections^21^. This option offers peace of mind and reduces the long-term risks associated with irreversible or permanent fillers. However, the popularity of HA fillers has also led to an increase in the number of inexperienced or unqualified practitioners offering these treatments^1^. The perception of HA fillers as a "safe" option may have contributed to a false sense of security among some providers and patients, leading to a higher incidence of adverse events^1^. Additionally, the temporary nature of HA fillers necessitates repeated treatments to maintain the desired effects, which may increase the cumulative risk of complications over time^22^.

The predominance of HA filler complications in our study highlights the need for stricter regulations on the qualifications of practitioners administering these treatments and for more comprehensive patient education on the potential risks associated with HA fillers. It also underscores the importance of proper technique, as even the most biocompatible and reversible fillers can lead to serious adverse events if not administered correctly.

Intriguingly, we observed a higher incidence of blindness in the left eye as compared to the right following filler injections. This laterality could stem from the predominance of right-handed injectors, whose technique may inadvertently favor the direction of filler towards the left eye. This discovery points to the critical need for practitioners to adopt proper injection techniques and to be acutely aware of how handedness might impact procedural outcomes.

This finding further emphasizes the importance of proper training and technique in minimizing the risk of complications associated with filler injections. Practitioners should be cognizant of their own handedness and take steps to ensure that their injection technique is consistent and safe, regardless of the side being treated. This may involve practicing injection techniques on both sides of the face during training, as well as being mindful of the angle and direction of the needle during the procedure itself.

Moreover, this laterality in blindness incidence underscores the need for a thorough understanding of facial anatomy and the potential pathways of filler emboli. By understanding the intricate network of blood vessels in the face and how they relate to the injection sites, practitioners can better avoid high-risk areas and minimize the chances of intravascular injection.

Among the most compelling of our findings is the substantial success rate of plaintiffs, especially in cases involving severe complications such as skin necrosis, blindness, and cerebral infarction. This underscores the paramount importance of adherence to standard care and informed consent in filler procedures. The courts' recognition of intravascular filler injection as negligence, even in the absence of direct evidence, highlights the need for medical professionals to exercise utmost caution and employ techniques to minimize the risk of vascular compromise, such as aspiration before injection, slow injection technique, and the use of blunt cannulas. In South Korea, the courts have established specific guidelines for filler injections, which include the use of local anesthetics with vasoconstrictors, aspiration before injection, slow injection technique, and the avoidance of high-risk areas (Supplemental sheet). These legal standards reflect the current best practices in aesthetic medicine and serve as a valuable reference for practitioners seeking to minimize the risk of complications and malpractice litigation.

This high success rate for plaintiffs underscores failures in informed consent processes, emphasizing the necessity for practitioners to thoroughly educate patients on potential risks. Patients should be fully aware of the potential risks and complications associated with filler injections, including the possibility of severe adverse events such as skin necrosis, blindness, and cerebral infarction. Practitioners should take the time to discuss these risks in detail with their patients and ensure that they understand the importance of seeking immediate medical attention if any signs of complications arise.

Furthermore, the medicolegal landscape surrounding filler complications underscores the need for robust training and certification programs for practitioners. Aesthetic medicine societies and regulatory bodies should work together to establish standardized curricula and certification requirements to ensure that all practitioners have the necessary skills and knowledge to perform filler injections safely and effectively.

Our analysis reveals substantial variability in the compensation awarded in different aesthetic medicine litigation cases. Specifically, we note that the total compensation awarded exhibits significant variation, with amounts ranging from 81,845,052 KRW (approximately 60,564 USD) to 208,813,674 KRW (roughly 154,518 USD) observed between cases adjudicated at the first and second instances. This variation not only underscores the complexity inherent in adjudicating such cases but also reaffirms the critical importance of informed consent and patient autonomy in aesthetic medicine practices. The recurrent provision of 'consolation money' in instances of informed consent violations points to the legal system's recognition of the fundamental nature of patient education and the shared decision-making process. These observations are congruent with international trends in medical jurisprudence that emphasize patient education as a pivotal factor in mitigating risks of adverse outcomes and reducing the incidence of malpractice litigation.

In comparison to prior studies on medical malpractice litigation in South Korea^6^, which have primarily focused on general medical practice or specific specialties such as plastic surgery, this study provides a targeted analysis of the legal issues surrounding cosmetic filler injections. The results corroborate and extend the findings of international studies^2,23,24^ that have documented the increasing incidence of filler complications and the associated medicolegal challenges.

South Korea's experience in dealing with cosmetic filler litigations offers valuable insights for the global medical community. As a leading market for aesthetic procedures, South Korea^25^ has witnessed a significant number of legal disputes related to filler complications. The high volume of cases provides a comprehensive dataset for understanding the challenges and potential solutions in this field.

While legal systems and regulatory frameworks may vary across countries, the core principles of medical negligence, informed consent, and patient safety remain universally applicable^26^. By examining the specific issues that have emerged in South Korea, such as the recognition of intravascular filler injection as negligence and the emphasis on comprehensive informed consent, other jurisdictions can identify areas for improvement in their own practices and policies.

The lessons learned from South Korea's experience can inform the development of international guidelines and best practices, contributing to the shared goal of enhancing patient safety and reducing legal disputes in aesthetic medicine worldwide. For instance, the South Korean courts' establishment of specific guidelines for filler injections, including the use of local anesthetics with vasoconstrictors, aspiration before injection, slow injection technique, and avoidance of high-risk areas, can serve as a reference point for other countries seeking to standardize their approach to filler treatments.

Moreover, the analysis of compensation trends and the impact of informed consent violations in South Korean cases can guide global efforts to improve patient education and shared decision-making processes. The substantial financial penalties associated with malpractice litigation in South Korea underscore the importance of prioritizing patient safety and adherence to ethical standards in aesthetic medicine.

However, it is essential to acknowledge that each country must adapt these insights to its unique legal, cultural, and healthcare contexts. While the overarching principles of patient rights, informed consent, and duty of care are fundamental to medical practice globally, the specific strategies for implementing these principles may vary depending on local factors such as healthcare access, cultural attitudes towards aesthetic procedures, and the maturity of the legal system^27^.

Nonetheless, the findings from South Korean cosmetic filler litigations provide a solid foundation for developing more robust and effective medical guidelines internationally. By leveraging the lessons learned from this significant market, the global medical community can work towards establishing a safer and more ethical environment for patients seeking aesthetic treatments worldwide.

From a legal perspective, the result of this study highlights the need for clearer guidelines on the standard of care and causation in filler-related lawsuits. The establishment of specialized courts or alternative dispute resolution mechanisms, such as mediation and arbitration, can help reduce the burden on the traditional court system and facilitate more timely and equitable resolution of claims. Moreover, the development of standardized expert witness guidelines and the promotion of interdisciplinary collaboration between medical and legal professionals can help ensure that legal proceedings are informed by the latest scientific evidence and best practices.

Turning our attention to the international landscape, a study by Beauvais and Ferneini analyzing malpractice litigation related to injectable facial fillers in the United States revealed that defendants prevailed in 6 out of 11 cases (54.5%)^23^. The awarded damages in these cases were substantial, ranging from $349,000 to $1,060,000. Interestingly, a lack of informed consent was alleged in 10 out of the 11 cases (90.9%) in the U.S. study, which echoes the high proportion of informed consent violations recognized in our study (81.8% in the first instance and 100% in the second instance) and the Brazilian study^14^. However, the success rates of plaintiffs in South Korea (68.2% in the first instance and 60% in the second instance) and Brazil (60% in the first instance and 43.8% in the second instance) were notably higher than that in the United States. This discrepancy may be attributed to differences in legal systems, medical practices, and societal perceptions of medical malpractice across these countries.

Furthermore, the U.S. study highlighted the role of non-physician injectors in filler-related litigation, with 50% of legal actions involving procedures performed by non-physicians. In contrast, our study and the Brazilian study primarily focused on cases involving physicians. This difference suggests that the scope of practice and regulations surrounding non-physician injectors may vary across countries and could impact the incidence and nature of filler-related lawsuits.

Despite these differences, the high prevalence of informed consent violations alleged in all three studies underscores the critical importance of a thorough and well-documented informed consent process in the context of cosmetic filler injections, regardless of the jurisdiction. The substantial financial penalties associated with these cases serve as a stark reminder of the costly repercussions of failing to provide adequate patient explanations. Clinicians attempting to capitalize on quick profits from fillers might find themselves paying significantly more in damages than the initial income from the procedures. This reinforces the necessity for clinicians to engage in detailed patient education and ensure understanding, thereby safeguarding both patient welfare and their professional practice.

These comparative findings underscore the global nature of the challenges posed by cosmetic filler complications and the importance of a multifaceted approach to risk management and patient care. By learning from the experiences and insights gained in different countries, practitioners and policymakers can work towards developing more effective strategies to promote the safe and ethical use of dermal fillers worldwide, emphasizing the need for continued research, education, and collaboration to ensure the highest standards of care for patients seeking these treatments.

In terms of public health, the increasing incidence of filler complications and the associated legal disputes underscore the need for greater regulation and oversight of the aesthetic medicine industry. Policymakers should consider implementing measures to ensure provider competency, product safety, and consumer protection, such as mandatory certification programs, adverse event reporting systems, and public awareness campaigns. Collaboration between regulatory agencies, professional societies, and patient advocacy groups can help promote evidence-based standards, enhance market transparency, and empower consumers to make informed decisions about their care.

This study has several limitations that should be acknowledged. The analysis relied on a sampling of court judgments available in the public domain, which may not capture the full spectrum of filler-related complications and disputes. The exclusion of unreported cases, out-of-court settlements, and cases handled through alternative dispute resolution mechanisms may have influenced the findings. Additionally, the retrospective nature of the study and the inherent subjectivity of qualitative analysis pose limitations on the interpretation of results.

Despite these limitations, the present study provides valuable insights into the legal aspects of cosmetic filler complications in South Korea. The analysis of court judgments sheds light on the types of complications, the factors influencing legal outcomes, and the measures deemed necessary by courts to prevent adverse events. These findings can inform clinical practice, policy development, and patient education initiatives, ultimately contributing to the improvement of patient safety and the reduction of legal disputes in the field of aesthetic medicine.

Future research should build upon the foundation laid by this study, aiming to address its limitations and expand the scope of investigation. Conducting prospective cohort studies could help establish the incidence and risk factors for filler complications, while longitudinal analyses could provide insights into the socioeconomic impact of filler-related legal disputes. Moreover, exploring the potential impact of technological advancements and investigating the psychosocial and ethical dimensions of cosmetic filler treatments could further contribute to the development of strategies for minimizing adverse events and improving patient outcomes in the aesthetic medicine industry.

The findings of this study underscore the fact that, under the current practice of filler injections, severe and irreversible complications arising from intravascular filler injections cannot be completely avoided. Such incidents are often recognized as a breach of the physician's duty of care. Consequently, it is imperative for practitioners to stay well-informed of the court-mandated precautionary measures and exercise the utmost caution during the injection process. In the event of complications, prompt actions may be necessary to mitigate liability.

Moreover, considering the high likelihood of substantial compensation payouts, seeking a reasonable settlement amount may prove to be a prudent strategy. The judgments in these cases tend to favor the patients, and unlike in the United States, physicians in South Korea are more likely to be held liable. In light of these findings, concerted efforts should be made to minimize the occurrence of complications caused by intravascular injections by leveraging advancements in technology. Furthermore, there is a pressing need for the development and implementation of social mechanisms and consensus to protect and support both patients and physicians in these unfortunate situations.

In summary, this study emphasizes the importance of practitioner awareness, adherence to precautionary measures, and the proactive management of complications. As the aesthetic medicine industry continues to evolve, it is crucial for all stakeholders—including practitioners, policymakers, and researchers—to collaborate in developing strategies that prioritize patient safety, minimize legal disputes, and foster a supportive environment for both patients and physicians. By doing so, we can work towards a future where the benefits of cosmetic filler treatments can be realized while mitigating the risks and challenges associated with these procedures.

### Supplementary Information

Supplementary Information.

## Data Availability

The data supporting the findings of this study are derived from publicly accessible court decisions indexed in the National Law Information Center^1^, “Big Case”^2^, and “Case Note”^3^. The search included terms related to cosmetic fillers and medical professionals, focusing on cases between 2007 and 2023 involving complications from filler injections by licensed practitioners. Cases involving unlicensed practices, additional treatments, insurance disputes, or procedural issues were excluded. Details on case selection and criteria are available in the methods section of the manuscript.
