# Supplementary material for: Legal analysis of South Korean cosmetic filler litigations for safer medical practices
Source: Sci Rep. 2024 Jun 10;14:13272. doi: 10.1038/s41598-024-63845-8 (PMC11164967; doi:10.1038/s41598-024-63845-8)
Supplement: Supplementary file 1 — Supplementary Information. [file 41598_2024_63845_MOESM1_ESM.docx]

**Supplemental Sheet: Legal Guidelines for Filler Injections in South Korea**

**Judicial Standards for Establishing Negligence in Filler Injection Procedures: Insights from South Korean Civil Litigation**

1. Injecting filler too deeply, beyond the subcutaneous layer, constitutes negligence.
2. Failure to perform regurgitation during filler injection is considered negligence.
3. Intravascular injection of filler is a violation of the duty of care and constitutes medical malpractice.
4. Discoloration, pain, or swelling at the injection site immediately after the procedure does not necessarily indicate vascular occlusion.
5. Failure to have filler-dissolving agents readily available may be considered joint illegal action or negligence.
6. Legal precautions for filler injections required by the courts:

A. Use local anesthetics containing vasoconstrictors.

B. Use flexible, fine needles appropriate for the injection site and material.

C. Before injecting the filler, confirm that the tip of the needle is not intravascular by performing regurgitation.

D. Inject the filler slowly with low pressure, avoiding excessive amounts by administering small quantities and adjusting the volume to prevent injecting large amounts in a narrow area.

E. Avoid injecting in areas where major vessels are located and do not inject the needle too deeply.

F. If symptoms such as headache or dizziness, or changes in skin color at the injection site occur during the injection process, immediately stop the injection and check for tissue necrosis.

G. In case of adverse events, inject filler-dissolving agents and provide emergency care, such as warm compresses and massage.

H. Additionally, consider administering vasodilators, diuretics, steroids, performing anterior chamber paracentesis to reduce intraocular pressure, or providing hyperbaric oxygen therapy. If a private clinic is unable to provide such treatments, the patient should be immediately transferred to a higher-level hospital after initial emergency care.

These guidelines, established by the courts in South Korean civil litigation, outline the standard of care expected from physicians during filler injection procedures. By adhering to these principles, practitioners can minimize the risk of complications and legal liability, ultimately promoting patient safety and well-being in the field of aesthetic medicine.
